# Fracture Resistance of CAD/CAM Implant-Supported 3Y-TZP-Zirconia Cantilevers: An In Vitro Study

**DOI:** 10.3390/ma15196638

**Published:** 2022-09-24

**Authors:** Mariana Novais, António Sérgio Silva, Joana Mendes, Pedro Barreiros, Carlos Aroso, José Manuel Mendes

**Affiliations:** UNIPRO–Oral Pathology and Rehabilitation Research Unit, University Institute of Health Sciences (IUCS), CESPU, 4585-116 Gandra, Portugal

**Keywords:** cantilever length, fracture, bite force, CAD/CAM, implant-supported prosthesis, zirconia

## Abstract

(1) Introduction: Implant-supported fixed complete dentures are mostly composed of cantilevers. The purpose of this work was to evaluate the fracture resistance of zirconia (Prettau^®^, second generation, or Ice Zirkon Translucent, first generation) with cantilever lengths of 6 and 10 mm, and zirconia’s fracture resistance in relation to an average bite force of 250 N. (2) Materials and methods: Forty structures were created in CAD/CAM and divided into four groups: group A (6 mm cantilever in IZT), group B (10 mm cantilever in IZT), group C (6 mm cantilever in Pz), and group D (10 mm cantilever in pz). The study consisted of a traditional “load-to-failure” test. (3) Results: A statistically significant result was found for the effect of cantilever length, t(38) = 16.23 (*p* < 0.001), with this having a large effect size, d = 4.68. The 6 mm cantilever length (M = 442.30, sd = 47.49) was associated with a higher mean force at break than the 10 mm length (M = 215.18, sd = 40.74). No significant effect was found for the type of zirconia: t(38) = 0.31 (*p* = 0.757), and d = 0.10. (4) Conclusions: All the components with cantilever lengths of 6 mm broke under forces higher than 250 N. Cantilevers larger than 10 mm should be avoided.

## 1. Introduction

The global elderly population will reach about 400 million in 2050, [1] which will increase partial or total edentulism. In the case of complete dentures, their stability and retention can be obtained through dental implants allowing fixed rehabilitation, or through the oral mucosa, in which dental adhesives, in the case of removable rehabilitation, play a fundamental role [2,3]. Fixed prostheses on implants are prostheses that have stability and retention, supported by dental implants, and they have been used for more than three decades. Osseointegrated dental implants have revolutionized prosthetic treatments and have become a fundamental alternative in the quality of life of populations [2,3,4,5]. However, implant-supported fixed rehabilitation can cause problems due to the anatomical and morphological conditions of the patient, which condition the selection and distribution of implants [2]. Computer-aided design and computer-aided manufacturing (CAD–CAM) can be used in different production techniques, namely, the subtractive milling and additive manufacturing. Regarding these two techniques, Valenti et al. showed that there is no significant difference between them in terms of the hardness, roughness, marginal discrepancy, fracture load, trueness, or internal fit. Furthermore, the additive manufacturing does not allow continuous mastication forces for a long period of time, being mostly used in provisional crowns and fixed partial dentures [6].

This is a determining factor in the design of prosthetic structures. Therefore, implant-supported fixed complete dentures, commonly known as hybrid prostheses, are mostly composed of cantilevers [7], that is, a multiple retainer with one or more unsupported free ends [8], thus allowing the prosthesis to extend to at least the first molar [2]. The use of cantilevers in fixed prostheses on dental implants is beneficial in places with unfavorable anatomical characteristics, namely, reduced alveolar ridges [9]; however, the placement of implants can be compromised due to their proximity to certain structures, such as the maxillary sinuses, the roots of adjacent teeth, and the inferior alveolar nerve [8]. However, long cantilevers should be avoided due to their biomechanical properties, which can lead to prosthesis fracture [5,7] as well as loss of bone around the implants [5]. As such, it is recommended that mandibular cantilevers do not exceed 20 mm, and ideally, they should be less than 15 mm in length [8]. Approximately half of patients rehabilitated with fixed prostheses containing cantilevers have long-term complications [2]. These can be due to high occlusal load, but also due to poorly distributed occlusal forces, which can lead to the loss or fracture of implants used to retain dental prostheses, which happens most often at the beginning of the arm of the cantilever. In order to avoid this type of fracture, shortening the mesiodistal, buccolingual/palatal length of the cantilever is recommended [2]. Thus, for a rehabilitation with implants to be successful, it is necessary that implant-supported prostheses are resistant to fracture [10].

Aesthetics plays a very important role in our society; as such, oral rehabilitation must take into account aesthetic requirements by using materials that meet the expectations of patients [11,12]. However, this should not compromise the strength, clinical success, and longevity of dental prostheses. The stress caused during their use can be transferred to the implants, to the bone, or to the supporting structures, which highlights the importance of the choice of material [13,14]. Zirconia differs from other materials due to the phenomenon called transformation hardening, with three pure forms of zirconia having been identified, depending on the temperature at which it is found [11].

Due to their aesthetic properties, there has been a substantial increase in the use of zirconia and ceramics in prosthetic rehabilitation [9,11,12]. This increase is due not only to the aesthetic component, but also due to these materials’ high fracture resistance and other mechanical properties [11], their excellent biocompatibility, and their physical properties [9,11,15]. These materials can be used in the elaboration of crowns, posts, fixed partial dentures, abutments, and structures on implants [9]. Currently, yttrium-stabilized tetragonal zirconia (Y-TZP) is the ceramic material that has the highest fracture resistance [16], and it is the ceramic material most used as a rehabilitation material in high stress areas due to its high strength [15]. It should be noted that today, the elaboration of zirconia structures is more optimized, accurate, and reliable, due to the development of computerized CAD–CAM systems [12,17,18,19,20]. They may be a viable alternative for full-arch implant-supported rehabilitations, being able to achieve high 5-year survival rates with minimal prosthetic complication rates since they were not subject to chipping and wear, even if work needs to be performed to improve their esthetics [21,22]. However, their medium- to long-term clinical outcomes cannot yet be evaluated [23,24,25].

Finite element analysis studies [26] showed that when force was applied over the cantilevers, the highest stress was concentrated in the distal posterior screw-access openings (SAOs), which is the zone with the minimum cross-sectional connector area (CSCA) because of the screw opening passing through it. This means that distal SAOs are the zones that are supposed to fracture when excessive strength is applied over the cantilever. This type of prosthesis distal extension fracture is directly related to the cantilever [27]. The other type of fractures are fractures, that occur between the distal SAOs and not involving them, are hypothesized to be more likely concerned with other causes, such as tension in the screw-retained structure, material defects, or excessive thinness of the zirconia framework [26,27].

An incidence of 5.6% prosthetic failure rate is described in most of the articles, but clinically we can observe a higher percentage that is not described [28].

Thus, this work has as its main objective to evaluate the fracture resistance of different cantilever lengths (CL) (6 and 10 mm). It also attempts to determine which of the zirconia forms (Prettau^®^ or Ice Zirkon Translucent) of lengths of 6 mm and 10 mm are more resistant to fracture, and to evaluate the fracture resistance of zirconia under an average bite force of 250 N. The null hypothesis tested is that the frameworks with a cantilever length of 6 mm have the same fracture force of the frameworks with a cantilever length of 10 mm. The limits of this study are those understood for an in vitro study and the application of force was performed only in one direction, excluding the application of oblique forces.

## 2. Materials and Methods

### 2.1. Materials

All materials used in this study were selected based on their importance and usefulness in dentistry, as well as their stability under normal conditions of use and storage. All materials and chemicals were used in accordance with manufacturers’ standards.

The materials used in this study were two different types of zirconia that belong to the same brand. One of the materials used was Ice Zirkon Translucent (IZT) (Zirkonzahn^®^, Gais, South Tyrol, Italy), and the other was Prettau^®^ Zirconia (PZ) (Zirkonzahn^®^, Gais, South Tyrol, Italy), as described in Table 1.

The interfaces used, as well as the screws, were from IPD^®^ (Mataró, Barcelona, Spain). These interfaces were selected because they are compatible with internal hexagon implants.

### 2.2. Methods

A standard laboratory protocol was established and applied at the Laboratório de Investigação em Reabilitação Oral e Prostodontia, UNIPRO—Oral Pathology and Rehabilitation Research Unit, University Institute of Health Sciences (IUCS), CESPU, Gandra, Portugal, to test all selected samples.

#### 2.2.1. Preparation of the Sample

**Figure 1:**
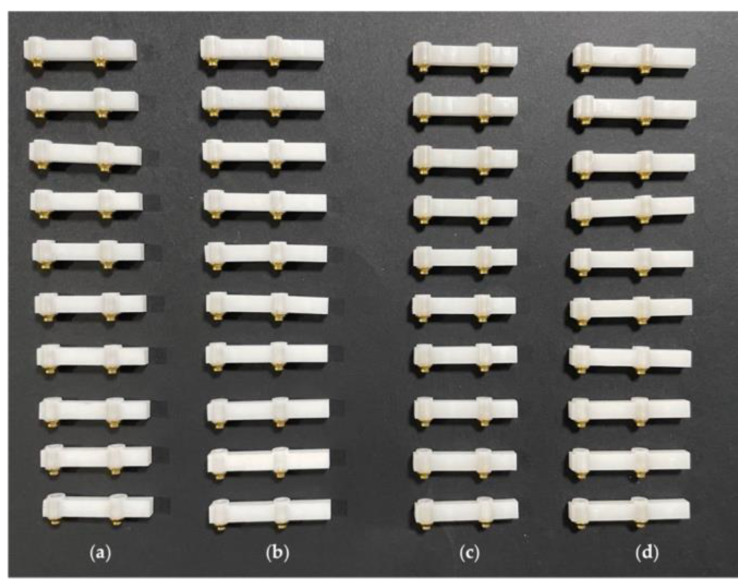
Study structures: (**a**) 6 mm Ice Zirkon Translucent cantilever; (**b**) 10 mm Ice Zirkon Translucent cantilever; (**c**) 6 mm Prettau^®^ Zirconia cantilever; (**d**) 10 mm Prettau^®^ Zirconia cantilever.

In this study, 40 CAD/CAM frameworks were prepared and divided into four groups: group A (10 frameworks with a 6 mm cantilever in Ice Zirkon Translucent), group B (10 frameworks with a 10 mm cantilever in Ice Zirkon Translucent), group C (10 frameworks with a 6 mm cantilever in Prettau^®^ Zirconia), and group D (10 frameworks with a 10 mm cantilever in Prettau^®^ Zirconia) (Figure 1). All the structures were implant-supported by two internal hexagon analogs with a 4.1 platform, and fixed on a titanium base, with there being a distance of 15 mm from their centers. They were all manufactured 4 mm high and 3 mm wide by IPD^®^ (Mataró, Barcelona, Spain). A titanium base was prepared so that it could be adapted to the support table to fix the testing machine, Instron^®^, Electropuls E10000 Linear-Torsion (Norwood, MA, USA), on which the two internal hexagon analogs with a 4.1 platform were coupled together.

The framework has a rectangle milled shape with linear polished edges.

#### 2.2.2. Elaboration of Zirconia Structures

**Figure 2:**
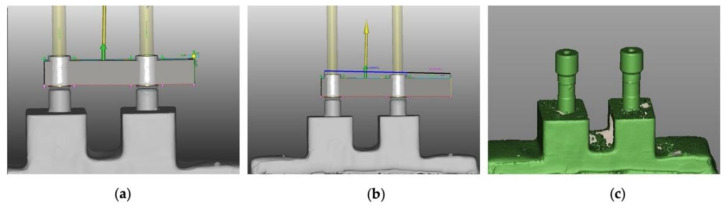
CAD/CAM elaboration of zirconia frameworks: (**a**) 6 mm cantilever; (**b**) 10 mm cantilever; (**c**) body scans.

The structures were digitally executed using a CAD/CAM system (Zirkonzahn^®^ Gais, South Tyrol, Italy) (Figure 2a,b). Two body scans (IPD^®^, Mataró, Barcelona, Spain) were placed on the base analogues and they were read in the Scanner S600 (Zirkonzahn^®^ Gais, South Tyrol, Italy) in order to be able to use a digital library and thus ensure that the structure would be well adapted to the interfaces (Figure 2c). Interfaces with a height of 3.5 mm were used.

After the structures were digitally executed, they were milled using the M1 machine from Zirkonzahn^®^ (Gais, South Tyrol, Italy). After this, they were removed from the zirconia blocks using a turbine and diamond drill, removing only the zirconia supports. The frameworks were sintered in a Zirkonofen 600 EV oven (Zirkonzahn^®^, Gais, South Tyrol, Italy). The IZT structures were placed in the oven at a maximum temperature of 1400 °C (gradual rise of 8 °C/min, 2 h at maximum temperature, and cooling of 8 °C/min until room temperature was reached), under which their dimensions contracted by 20%. In turn, the PZ structures were placed in the oven at a maximum temperature of 1600 °C (gradual rise of 6 °C/min, 2 h at maximum temperature, and cooling of 6 °C/min until room temperature was reached), under which they contracted in size by 19.95%.

In order to complete the test structures, the interfaces were screwed to the base (previously prepared) at 35 N using a torque wrench. The structures were cemented to the interfaces with Maxcem EliteTM resin cement (Kerr^®^, Kloten, Switzerland), following the manufacturer’s instructions. Teflon tape was placed in the access channel to the interface screws to prevent the entry of cement. After polymerization, all excesses were removed.

#### 2.2.3. Compression Test to Measure the Fracture Resistance Strength of Different Cantilevers

The titanium base, with the implant analogs made for the study, was attached to the Instron^®^ fixation support table, as shown, allowing its connection to the Instron^®^ testing machine, the Electropuls E10000 Linear-Torsion.

The Instron^®^ Electropuls E10000 LT is a dynamic and fatigue testing machine with linear dynamic capacity of ±10 KN, linear static capacity of ±7 KN, linear stroke of 60 mm, torque capacity of ±100 Nm, torsion stroke of ±135°, and daylight 877 mm opening, which allows both static and dynamic axial and torsional tests, in accordance with ISO 7500-1:2018. It has a calibration accredited force according to ISO 7500-1 and ASTM E4 standard of up to 5 meganewtons. The fixture support table was attached to the machine to adapt the simulation structures, so that all the models were adjusted and produced equal compression. In this way, the test structures were parallel to the transport table.

**Figure 3:**
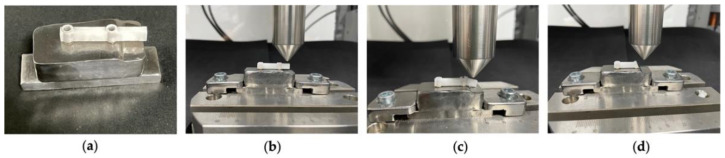
Fracture resistance tests. (**a**) Titanium-based bolted structure; (**b**) start of the static load, 2 mm from the end of the cantilever arm; (**c**) fracture of the 6 mm cantilever; (**d**) fracture of the 10 mm cantilever.

The test structures (bars with cantilevers and interfaces) were screwed to the titanium base, as shown in Figure 3a, at 35 N using a torque wrench. The study consisted of a traditional load-to-failure test in which a static load was used, 2 mm from the end of the cantilever arm (Figure 3b), which progressively increased by 1 mm/min towards the structure until the fracture occurred (Figure 3c,d), as described in Alshiddi et al.’s study [9].

The number of samples that was used for each zirconia type was 10 replicates (10 cantilevered frameworks) with 6 mm and 10 mm cantilever lengths. In this way, the fracture resistance force of each type of structure was measured 10 times, and the values were recorded in newtons (N).

Test results were transferred to WaveMatrix^®^ version 2.0 Dynamic Test Software (Instron^®^, Norwood, MA, USA). This software allows users to define and run tests and acquire data for a wide variety of dynamic and quasi-static applications. Then, all the values and data were transferred to Microsoft Office Excel^®^, version 16.0 (Redmond, WA, USA) where the statistical analysis of the obtained data was performed.

### 2.3. Statistical Analysis

Data were analyzed with R, version 4.1.2 (R Core Team, R Foundation for Statistical Computing, Vienna, Austria). Initial data inspection included normality assessment of force at break (standard) measured in newtons (N), using the Shapiro–Wilk test, which is appropriate for sample sizes lower than 50. Levene’s test was used to assess the homogeneity of variance. Considering the results of the tests, parametric tests were implemented. Descriptive statistics are presented as means (M) and standard deviations (SD). Boxplots were also used to plot the distribution of force at break, with points added that corresponded to the sampling observations. Observation overlap was avoided by including a jitter function. Independent samples *t*-tests were used to compare force at break according to the type of zirconia (Translucent and Prettau), cantilever length (6 mm and 10 mm), and the type of zirconia stratified by each cantilever length. Cohen’s d was calculated for effect size, with 0.2 considered a small effect size, 0.5 a medium effect size, and 0.8 a large effect size.

The interaction between the type of zirconia and cantilever length was assessed using factorial ANOVA. Partial eta squared (η_p_^2^) was used to assess effect size, with 0.01 considered a small effect size, 0.06 a medium effect size, and 0.14 a large effect size

One-sample *t*-tests were used to compare the mean forces at break under the limit of 250 N.

Statistical significance was considered for *p* < 0.05. A higher threshold (marginal significance, *p* < 0.10) was considered when the sample size was 10 or lower.

## 3. Results

**Figure 4:**
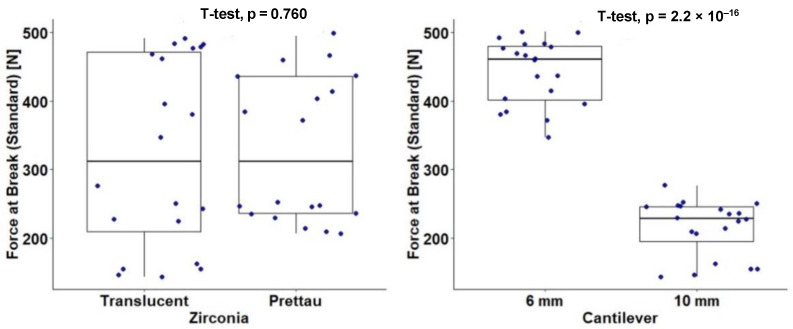
Force at break (standard) (N) according to type of zirconia and cantilever length.

A total of 40 component samples were enrolled in a 2 × 2 design that considered the type of zirconia (Translucent and Prettau) and the cantilever length (6 mm and 10 mm). The resistance to fracture was assessed with the force at break (standard) measured in newtons (N). Table 2 and Figure 4 show results for the independent effects of the type of zirconia and cantilever length on force at break. A statistically significant result was found for the effect of cantilever length, t(38) = 16.23 (*p* < 0.001), with this having a large effect size, d = 4.68. Lengths of 6 mm (M = 442.30, sd = 47.49) were associated with a higher mean force at break when compared with lengths of 10 mm (M = 215.18, sd = 40.74). No significant effect was found for the zirconia type: t(38) = 0.31 (*p* = 0.757), d = 0.10.

**Figure 5:**
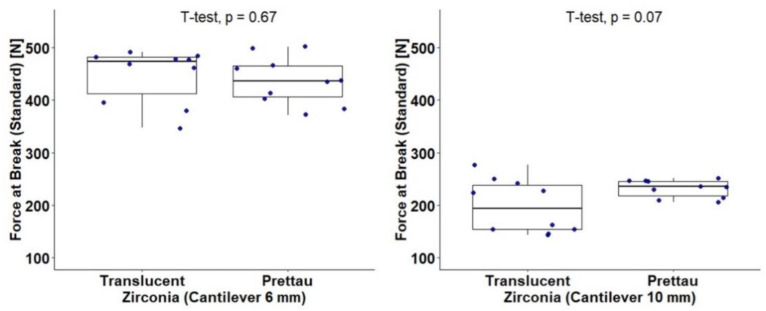
Force at break (standard) (N) according to type of zirconia stratified by cantilever length (6 mm and 10 mm).

Table 3 and Figure 5 show results for the independent effect of the type of zirconia stratified by cantilever length (6 mm and 10 mm). For the 10 mm length, a marginal effect was detected, t(18) = −2.01 (*p* = 0.070), with this having a large effect size, d = 0.90. The Prettau Zirconia components showed a higher mean force at break (M = 232.18, sd = 16.83) than the Ice Zirkon Translucent components (M = 198.18, sd = 50.79). No marginal or significant effect was detected for the 6 mm cantilever length.

**Figure 6:**
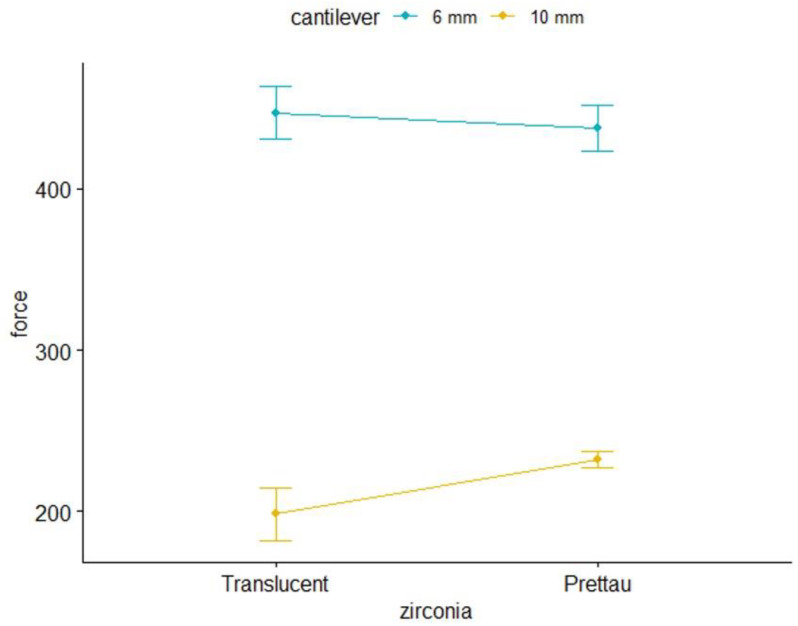
Force at break (standard) (N) interaction between type of zirconia and cantilever length.

Next, the interaction between the type of zirconia and cantilever length was studied by conducting a factorial ANOVA (Table 4, Figure 6). The initial effects of the type of zirconia and cantilever length were assessed. Cantilever length showed a significant effect, F_(1,36)_ = 272.46 (*p* < 0.001), a large effect size, η_p_^2^ = 0.88, and a higher mean force at break for 6 mm, as previously shown. No significant effect was found for the type of zirconia: F_(1,36)_ = 0.79 (*p* = 0.379), and η_p_^2^ = 0.02. The interaction between the type of zirconia and cantilever length was also not statistically significant: F_(1,36)_ = 2.50 (*p* = 0.273), and η_p_^2^ = 0.07, meaning that the cantilever length effect of 6 mm was independent of the type of zirconia.

Finally, force at break was compared to the limit of 250 N under the null hypothesis of H_0_: μ ≤ 250 N vs. the alternative of H_1_: μ > 250 N. Comparisons were made that considered all samples as a whole and by group (Table 5). Unilateral one-sample *t*-tests showed statistically significant differences (*p* < 0.001) for all samples of a 6 mm cantilever length, regardless of the zirconia type, indicating that the required force to break the components is higher than 250 N for components with a cantilever length of 6 mm. All components with a 6 mm cantilever length broke under forces higher than 250 N. On the contrary, for the cantilever length of 10 mm, the null hypothesis was not rejected (*p* > 0.999) for both Ice Zirkon Translucent, with just two (20%) components of which broke under forces higher than 250 N, and Prettau Zirconia, with just 1 (10%) component of which broke after applying forces higher than 250 N.

**Figure 7:**
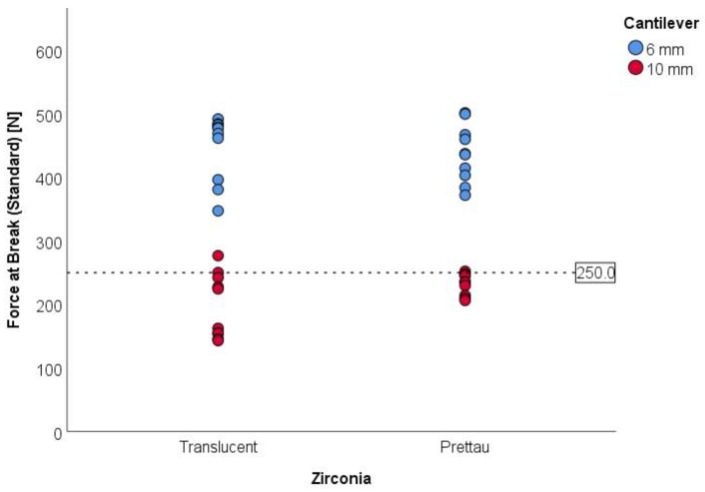
Force at break (standard) (N) for all components included in the study and the considered limits.

Figure 7 shows that all the components included in the study needed to be submitted to forces higher than 250 N to break. As previously shown, all components with a cantilever length of 6 mm required a force higher than 250 N to break. In the whole sample, the minimum and maximum forces at break were 143.07 N and 501.58 N, respectively.

## 4. Discussion

For fixed implant rehabilitations, namely, rehabilitations with structures with cantilevers, the CL is a very important factor to take into account, as it influences the longevity of the rehabilitation. In this study, we tested the fracture resistance of different lengths of cantilevers, 6 and 10 mm, and also the resistance of two different types of zirconia, Ice Zirkon Translucent and Prettau^®^ Zirconia. For this purpose, 40 frameworks were made and tested: 20 frameworks with a 6 mm cantilever, of which 10 were in Ice Zirkon Translucent and 10 were in Prettau^®^ Zirconia, and 20 frameworks with a 10 mm cantilever, of which 10 were also in Ice Zirkon Translucent and in Prettau^®^ zirconia.

Tirone et al. reported on the CL or the thickness of the zirconia around the screw access opening (SAO) to the fracture of the structure [29], and their results were in line with those found in our study, because the SAO was the zone where there was a fracture in the zirconia structures. In order to overcome this problem, Alshiddi et al. used a thickness increase of 0.5 mm around the distal SAO in order to reinforce it [9]. In our study, the value used around the SAO was the predefined value of the CAD/CAM program, namely, 0.5 mm, in order not to modify the values that are established for use in the elaboration of structures of implant-supported zirconia prostheses.

With regard to CL, the results obtained in this study corroborate what is described by several authors [2,9,29]. Bearing in mind that the ideal cantilever length should not exceed 9 mm, in this study, structures with 6 mm and 10 mm cantilevers were tested in order to test their resistance to fracture. A higher force was required to cause fracture in 6 mm structures (a mean force of 442.30 N) than in 10 mm structures (a mean force of 215.18 N). It was also found that when a force was applied to the cantilever, the greatest stress point was concentrated in the distal SAO, with there being a smaller cross-sectional connector area due to access to the SAO, these being the areas where fracture is expected.

Bite force (BF) is an important component of mastication as well as masticatory function [30]. The measurement of BF allows us to determine the average BF values of a given population, thus helping us choose the most suitable and resistant materials during prosthetic rehabilitation. Based on Takaki et al.’s study, in which they reported a mean BF of 285.01 N for women and 253.99 N for men [31], and Levartovsky et al.’s study, in which they reported an average BF of between 258.5 N and 175.8 N [32], we used an average BF reference value of 250 N. Taking into account this average BF value and analyzing the results obtained, we can see that all structures with a 6 mm cantilever required a force greater than the average reference value for fracture to occur, unlike structures with a 10 mm cantilever, of which only three structures resisted forces greater than 250 N (one in PT and two in IZT). However, Van Vuuren et al. reported an average BF of 430.4 N [33], and, taking this value into account, we can see that only structures with a 6 mm cantilever can resist this average BF, given that the mean fracture values found in this study were 437.54 N in PT and 447.05 N in IZT. Taking into account these mean BF values and based on the results of this study, we can say that in terms of longevity of prosthetic rehabilitations, structures with 6 mm cantilevers have a higher fracture resistance behavior than those found in cantilevers 10 mm long. Regarding the type of zirconia used (Ice Zirkon Translucent and Prettau^®^ Zirconia), they performed similarly in terms of fracture resistance, with there being no statistically significant differences between them.

Y-TZP, a widely developed ceramic rehabilitation material [34], is considered the most resistant and robust ceramic material available [35]. Clinically, stratified crowns after prolonged use have shown fissures or fractures, leading to rehabilitation failure [12,36,37]. This problem has been solved with the introduction and development of monolithic zirconia, which is manufactured in CAD/CAM. The first generation of 3Y-TZP was developed to be the strongest, accepting loads of up to 1200 MPa [24]. In this type of rehabilitation, all the ceramic layering is removed, thus preventing one of the reasons for its failure [34,36,37].

In this study, the differences in the average strength of materials were not considered significant. IZT presented an average fracture resistance of 322.62 N, while PZ presented an average fracture resistance of 334.86 N. However, even though the difference in the strength of the different materials was minimal, the fracture strength of the 10 mm cantilever PZ obtained better results than that of the IZT, the former requiring an average force of 232.18 N compared to the latter’s 198.18 N. Meanwhile, the 6 mm cantilever PZ required an average force of 437.54, and the IZT required an average force of 447.05 N.

The framework shape was not evaluated in this study, but some studies suggest that geometry influences the material behavior and fracture, e.g., a conventional shape (linear, e.g., our framework design); convex shape (1.0 mm curve in the direction of the occlusal surface); concave shape (1.0 mm curve in the direction of the gingival surface). The different results, for example, in Tsumita et al.’s study of the mechanical strength in different framework shapes, showed that the fracture load for the convex shape was the highest; however, critical cracks in the veneer porcelain were seen in the convex shape, but not in the other two shapes [38]. These cracks occurred from the lower margin of the pontic framework towards the gingival surface of the medial and distal connectors of the pontic framework. Such failure is not clinically acceptable. Because of the geometry of the convex shape, it was difficult for the frameworks located on the gingival side of the connector where stress concentrates; thus, the veneer porcelain received the tensile stress directly, and cracks were initiated at a low load value. In addition, the final fracture load was high, because the shape of frameworks resembled a reverse catenary, and thus received the loading stress as compressive stress. The fracture load for the concave shape was significantly higher than that for the conventional shape; the veneer porcelain cracking load for the concave shape was significantly greater than that for the convex shape. In terms of bridge engineering, the concave shape resembled a catenary; however, by arranging a framework without a pontic–connector interface where stress concentrates in an area of maximum principal stress, the load could be evenly dispersed throughout the lower margin of the frame.

Conventionally cast noble (gold/silver–palladium) or not noble (Co–Cr) metal alloys are the most traditionally employed materials for full-arch implant-supported rehabilitations, reporting high clinical performances with optimal clinical implant and prosthetic survival rates in the long term. However, various alternative materials are available today, such as titanium, zirconia, and several polymers including carbon-fiber frameworks, providing corrosion resistance, biocompatibility, and great mechanical characteristics, with satisfactory clinical outcomes [28].

Further comparative clinical studies, possibly randomized clinical trials with a longer follow-up time, are needed in order to validate the use of new materials and define their specific clinical indications.

## 5. Conclusions

In the present study, it was found that there was a big difference in the fracture resistance of the cantilevers used, and, taking into account the average BF, fracture strength was much higher in the 6 mm cantilevers compared to the 10 mm cantilevers.

Regarding the type of zirconia, IZT or PT, we found that there were no statistically significant differences regarding their strength in the two cantilever lengths (6 and 10 mm).

Taking into account the average BF of 250 N, in the 6 mm cantilever, the IZT and PT did not show significant differences in fracture resistance, both presenting an average fracture force greater than 250 N. On the other hand, in the 10 mm cantilevers, the IZT and the PT obtained inferior results in relation to this mean BF value in terms of fracture resistance, with both presenting a lower mean fracture force.

## Figures and Tables

**Table 1:** Zirconia characteristics.

Zirconia	Flexural Strength	Vickers Hardness (HV10)
Ice Zirkon Translucent	1200–1400 MPa	1250 HV10
Prettau^®^ Zirconia	1000–1200 MPa	1250 HV10

**Table 2:** Force at break (standard) (N) according to type of zirconia and cantilever length.

	Type of Zirconia			Cantilever Length		
	Translucent (n = 20)	Prettau (n = 20)	*t*-Test	6 mm (n = 20)	10 mm (n = 20)	*t*-Test
Force at Break (Standard) (N)	322.62 (137.09)	334.86 (110.41)	t(38) = 0.31 (*p* = 0.757) d = 0.10	442.30 (47.49)	215.18 (40.74)	t(38) = 16.23 (*p* < 0.001) d = 4.68

**Table 3:** Force at break (standard) (N) according to type of zirconia stratified by cantilever length (6 mm and 10 mm).

	Cantilever Length = 6 mm (n = 20)			Cantilever Length = 10 mm (n = 20)		
	Translucent Zirconia (n = 10)	Prettau Zirconia (n = 10)	*t*-Test	Translucent Zirconia (n = 10)	Prettau Zirconia (n = 10)	*t*-Test
Force at Break (Standard) (N)	447.05 (51.84)	437.54 (44.97)	t(18) = 0.44 (*p* = 0.667) d = 0.20	198.18 (50.79)	232.18 (16.83)	t(18) = −2.01 (*p* = 0.070) ^‡^ d = 0.90

^‡^*p* < 0.10.

**Table 4:** Factorial ANOVA for the effect of type of zirconia and cantilever length on force at break (standard) (N).

		n	M	SD	Factorial ANOVA
Zirconia	Cantilever				
Translucent	6 mm	10	447.05	51.84	Zirconia effect F_(1,36)_ = 0.79 (*p* = 0.379), η_p_^2^ = 0.02
	10 mm	10	198.18	50.79	
	Total	20	322.62	137.09	
Prettau	6 mm	10	437.54	44.97	Cantilever effect F_(1,36)_ = 272.46 (*p* < 0.001), η_p_^2^ = 0.88
	10 mm	10	232.18	16.83	
	Total	20	334.86	110.41	
Total	6 mm	20	442.30	47.49	Zirconia x Cantilever effect F_(1,36)_ = 2.50 (*p* = 0.273), η_p_^2^ = 0.07
	10 mm	20	215.18	40.74	
	Total	40	328.74	123.02	

**Table 5:** Unilateral one-sample *t*-tests for assessing limits of force at break (standard) (N).

		Tests for Mean > 250 N				Proportion of Components > 250 N
		n	M	SD	*t*-Tests	n (%)
Zirconia	Cantilever					
Translucent	6 mm	10	447.05	51.84	t(9) = 12.02 (*p* < 0.001)	10 (100%)
	10 mm	10	198.18	50.79	t(9) = −3.23 (*p* > 0.999)	2 (20%)
	Total	20	322.62	137.09	t(19) = 2.37 (*p* = 0.015)	12 (60.0%)
Prettau	6 mm	10	437.54	44.97	t(9) = 13.19 (*p* < 0.001)	10 (100%)
	10 mm	10	232.18	16.83	t(9) = −3.35 (*p* > 0.999)	1 (10%)
	Total	20	334.86	110.41	t(19) = 3.44 (*p* = 0.002)	11 (55.0%)
Total	6 mm	20	442.30	47.49	t(19) = 18.11 (*p* < 0.001)	20 (100%)
	10 mm	20	215.18	40.74	t(19) = −3.82 (*p* > 0.999)	3 (15.0%)
	Total	40	328.74	123.02	t(39) = 4.05 (*p* < 0.001)	23 (57.5%)

## Data Availability

The data that support the findings of this study are available from the corresponding author upon request.

## References

[1] Petersen P.E., Kandelman D., Arpin S., Ogawa H. (2010). Global oral health of older people–call for public health action. Community Dent Health.

[2] Minoretti R., Triaca A., Saulacic N. (2012). Unconventional implants for distal cantilever fixed full-arch prostheses: A long-term evaluation of four cases. Int. J. Periodontics Restor. Dent..

[3] Mendes J., Mendes J.M., Barreiros P., Aroso C., Silva A.S. (2022). Retention Capacity of Original Denture Adhesives and White Brands for Conventional Complete Dentures: An In Vitro Study. Polymers.

[4] Takaba M., Tanaka S., Ishiura Y., Baba K. (2013). Implant-supported fixed dental prostheses with CAD/CAM-fabricated porcelain crown and zirconia-based framework. J. Prosthodont..

[5] Gonçalves F.C.P., Amaral M., Borges A.L.S., Gonçalves L.F.M., Paes-Junior T.J.A. (2018). Fracture load of complete-arch implant-supported prostheses reinforced with nylon-silica mesh: An in vitro study. J. Prosthet. Dent..

[6] Valenti C., Isabella F.M., Masciotti F., Marinucci L., Xhimitiku I., Cianetti S., Pagano S. (2022). Mechanical properties of 3D-printed prosthetic materials compared with milled and conventional processing: A systematic review and meta-analysis of in vitro studies [published online ahead of print, 2022 Aug 5]. J. Prosthet. Dent..

[7] Shackleton J.L., Carr L., Slabbert J.C., Becker P.J. (1994). Survival of fixed implant-supported prostheses related to cantilever lengths. J. Prosthet. Dent..

[8] Chong K.K., Palamara J., Wong R.H., Judge R.B. (2014). Fracture force of cantilevered zirconia frameworks: An in vitro study. J. Prosthet. Dent..

[9] Alshiddi I.F., Habib S.R., Zafar M.S., Bajunaid S., Labban N., Alsarhan M. (2021). Fracture Load of CAD/CAM Fabricated Cantilever Implant-Supported Zirconia Framework: An In Vitro Study. Molecules.

[10] Honda J., Komine F., Kusaba K., Kitani J., Matsushima K., Matsumura H. (2020). Fracture loads of screw-retained implant-supported zirconia prostheses after thermal and mechanical stress. J. Prosthodont. Res..

[11] Alraheam I.A., Donovan T., Boushell L., Cook R., Ritter A.V., Sulaiman T.A. (2020). Fracture load of two thicknesses of different zirconia types after fatiguing and thermocycling. J. Prosthet. Dent..

[12] Fischer P., Barbu H.M., Fischer C.A.I., Pantea M., Baciu F., Vranceanu D.M., Cotrut C.M., Spinu T.C. (2021). Bending Fracture of Different Zirconia-Based Bioceramics for Dental Applications: A Comparative Study. Materials.

[13] Bacchi A., Consani R.L., Mesquita M.F., Dos Santos M.B. (2013). Effect of framework material and vertical misfit on stress distribution in implant-supported partial prosthesis under load application: 3-D finite element analysis. Acta Odontol. Scand..

[14] Ferreira M.B., Barão V.A., Faverani L.P., Hipólito A.C., Assunção W.G. (2014). The role of superstructure material on the stress distribution in mandibular full-arch implant-supported fixed dentures. A CT-based 3D-FEA. Mater. Sci. Eng. C Mater. Biol. Appl..

[15] Hafezeqoran A., Koodaryan R., Hemmati Y., Akbarzadeh A. (2020). Effect of connector size and design on the fracture resistance of monolithic zirconia fixed dental prosthesis. J. Dent. Res. Dent. Clin. Dent. Prospect..

[16] Schriwer C., Skjold A., Gjerdet N.R., Øilo M. (2017). Monolithic zirconia dental crowns. Internal fit, margin quality, fracture mode and load at fracture. Dent. Mater..

[17] Chougule K.J., Wadkar A.P. (2017). An In vitro Comparative Evaluation of Flexural Strength of Monolithic Zirconia after Surface Alteration Utilising Two Different Techniques. J. Clin. Diagn. Res..

[18] Juntavee N., Juntavee A., Phattharasophachai T. (2021). Fracture toughness of different monolithic zirconia upon post-sintering processes. J. Clin. Exp. Dent..

[19] Chang Y.T., Wu Y.L., Chen H.S., Tsai M.H., Chang C.C., Wu A.Y. (2022). Comparing the Fracture Resistance and Modes of Failure in Different Types of CAD/CAM Zirconia Abutments with Internal Hexagonal Implants: An In Vitro Study. Materials.

[20] The R Development Core Team (2021). A Language and Environment for Statistical Computing. http://softlibre.unizar.es/manuales/aplicaciones/r/fullrefman.pdf.

[21] Bidra A.S., Tischler M., Patch C. (2018). Survival of 2039 complete arch fixed implantsupported zirconia prostheses: A retrospective study. J. Prosthet. Dent..

[22] Tischler M., Patch C., Bidra A.S. (2018). Rehabilitation of edentulous jaws with zirconia complete-arch fixed implant-supported prostheses: An up to 4-year retrospective clinical study. J. Prosthet. Dent..

[23] Poggio C.E., Ercoli C., Rispoli L., Maiorana C., Esposito M. (2017). Metal-free materials for fixed prosthodontic restorations. Cochrane Database Syst. Rev..

[24] Bidra A.S., Rungruanganunt P., Gauthier M. (2017). Clinical outcomes of full arch fixed implant-supported zirconia prostheses: A systematic review. Eur. J. Oral Implantol..

[25] Abdulmajeed A.A., Lim K.G., Närhi T.O., Cooper L.F. (2016). Complete-arch implant-supported monolithic zirconia fixed dental prostheses: A systematic review. J. Prosthet. Dent..

[26] Ozan O., Kurtulmus-Yilmaz S. (2018). Biomechanical comparison of different implant inclinations and cantilever lengths in all-on-4 treatment concept by three-dimensional finite element analysis. Int. J. Oral Maxillofac. Implants.

[27] Alshahrani F.A., Yilmaz B., Seidt J.D., McGlumphy E.A., Brantley W.A. (2017). A load-to-fracture and strain analysis of monolithic zirconia cantilevered frameworks. J. Prosthet. Dent..

[28] Delucchi F., De Giovanni E., Pesce P., Bagnasco F., Pera F., Baldi D., Menini M. (2021). Framework Materials for Full-Arch Implant-Supported Rehabilitations: A Systematic Review of Clinical Studies. Materials.

[29] Tirone F., Salzano S., Rolando E., Pozzatti L., Rodi D. (2022). Framework Fracture of Zirconia Supported Full Arch Implant Rehabilitation: A Retrospective Evaluation of Cantilever Length and Distal Cross-Sectional Connection Area in 140 Patients Over an Up-To-7 Year Follow-Up Period. J. Prosthodont..

[30] Al-Omiri M.K., Sghaireen M.G., Alhijawi M.M., Alzoubi I.A., Lynch C.D., Lynch E. (2014). Maximum bite force following unilateral implant-supported prosthetic treatment: Within-subject comparison to opposite dentate side. J. Oral Rehabil..

[31] Takaki P., Vieira M., Bommarito S. (2014). Maximum bite force analysis in different age groups. Int. Arch. Otorhinolaryngol..

[32] Levartovsky S., Peleg G., Matalon S., Tsesis I., Rosen E. (2022). Maximal Bite Force Measured via Digital Bite Force Transducer in Subjects with or without Dental Implants—A Pilot Study. Appl. Sci..

[33] van Vuuren L.J., Broadbent J., Duncan W., Waddell J. (2020). Maximum voluntary bite force, occlusal contact points and associated stresses on posterior teeth. J. R. Soc. N. Z..

[34] Schönhoff L.M., Lümkemann N., Buser R., Hampe R., Stawarczyk B. (2021). Fatigue resistance of monolithic strength-gradient zirconia materials. J. Mech. Behav. Biomed. Mater..

[35] Zhang Y., Lawn B.R. (2018). Novel Zirconia Materials in Dentistry. J. Dent. Res..

[36] Tang Z., Zhao X., Wang H., Liu B. (2019). Clinical evaluation of monolithic zirconia crowns for posterior teeth restorations. Medicine.

[37] Pjetursson B.E., Sailer I., Latyshev A., Rabel K., Kohal R.J., Karasan D. (2021). A systematic review and meta-analysis evaluating the survival, the failure, and the complication rates of veneered and monolithic all-ceramic implant-supported single crowns. Clin. Oral Implants Res..

[38] Tsumita M., Kokubo Y., Steyern P.V., Fukushima S. (2008). Effect of Framework Shape on the Fracture Strength of Implant-Supported All-Ceramic Fixed Partial Dentures in the Molar Region. J. Prosthodont..

